# Correction: Blockade of the LRP16-PKR-NF-κB signaling axis sensitizes colorectal carcinoma cells to DNA-damaging cytotoxic therapy

**DOI:** 10.7554/eLife.57666

**Published:** 2020-04-16

**Authors:** Xiaolei Li, Zhiqiang Wu, Xiaojing An, Qian Mei, Miaomiao Bai, Leena Hanski, Xiang Li, Tero Ahola, Weidong Han

Li X, Wu Z, An X, Mei Q, Bai M, Hanski L, Li X, Ahola T, Han W. 2017. Blockade of the LRP16-PKR-NF-κB signaling axis sensitizes colorectal carcinoma cells to DNA-damaging cytotoxic therapy. *eLife* **6**:e27301. doi: 10.7554/eLife.27301.

Published 18, August 2017

Due to an oversight at the time of the manuscript publication, several of the images in Figure 6G and Figure 2—figure supplement 2B were unintentionally duplicated or overlapping, a mistake that was brought to our attention by a reader who had read the published version of the article. Also, we recently noticed that the image within Figure 2—figure supplement 3A was mistakenly picked.

Specifically, in Figure 6G the images of the cell areas in the first row (left and middle) were duplicated in error and in the second row (leftmost) and third row (rightmost) we mistakenly picked two images showing overlapping cell areas. Also, in Figure 2-figure supplement 2B we unintentionally duplicated the two images showing the results for the 1μg/ml oxaliplatin concentrations and in the first row (leftmost) we mistakenly picked image showing the result for the 0μg/ml oxaliplatin concentrations. In Figure 2—figure supplement 3A the image of the cell area in the third row (rightmost) was mistakenly picked. These errors occurred inadvertently during conversion of figure file formats and subsequent figure rearrangement. The correction does not affect or change any of the conclusions of the manuscript.

Additionally, the figure legends of Figure 3—figure supplement 2 and Figure 3—figure supplement 3 in the published version should be interchanged.

The Corrected Figure 6G is shown here:

**Figure 1:**
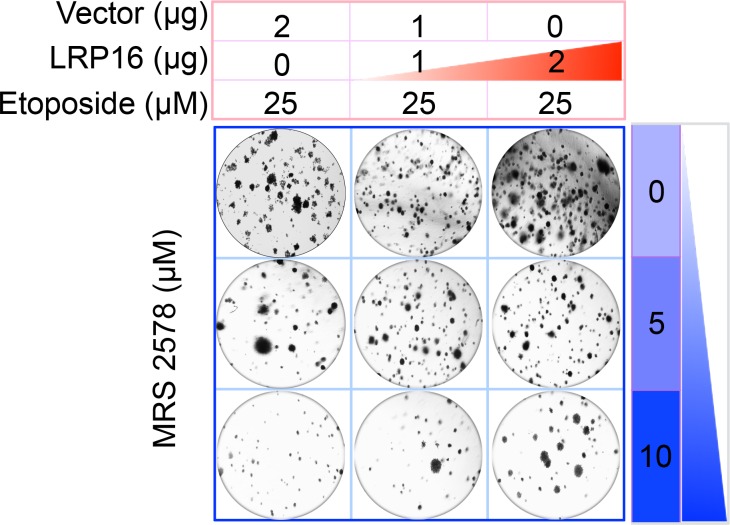


The originally published Figure 6G is also shown for reference:

**Figure 2:**
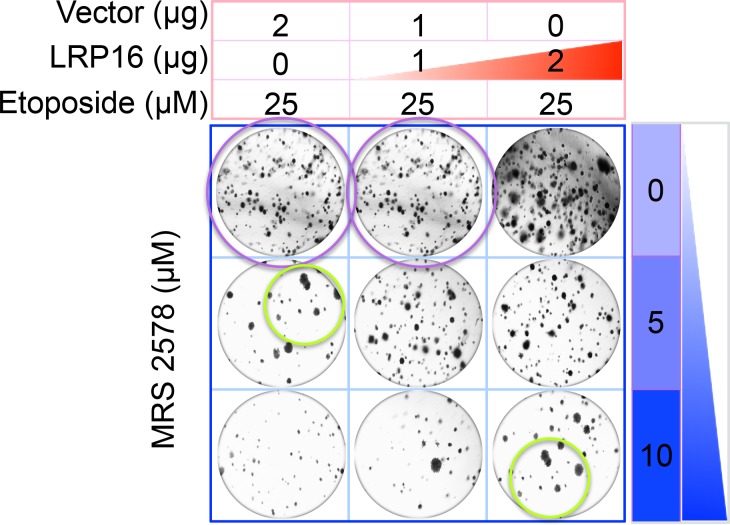


The Corrected Figure 2—figure supplement 2B is shown here:

**Figure 3:**
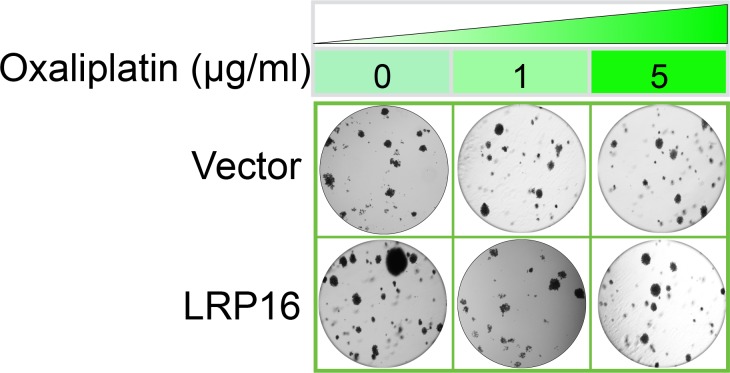


The originally published Figure 2—figure supplement 2B is also shown for reference:

**Figure 4:**
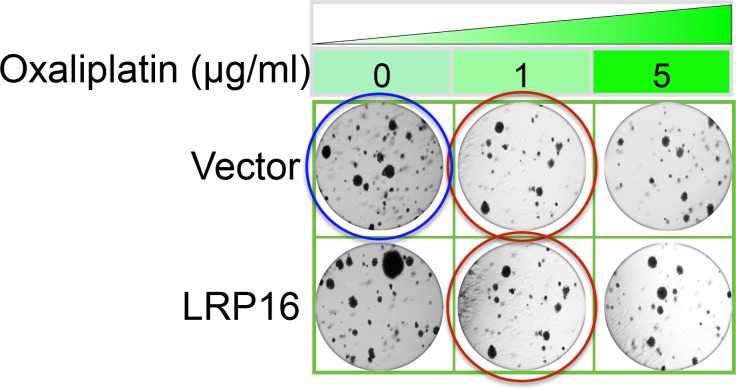


The Corrected Figure 2—figure supplement 3A is shown here:

**Figure 5:**
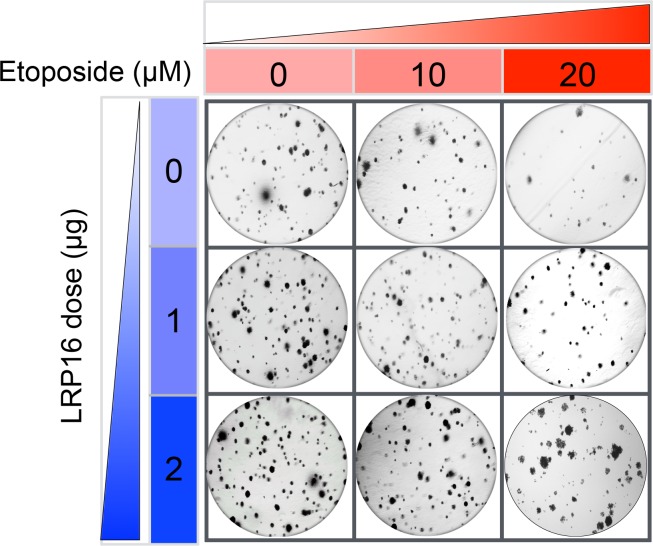


The originally published Figure 2—figure supplement 3A is also shown for reference:

**Figure 6:**
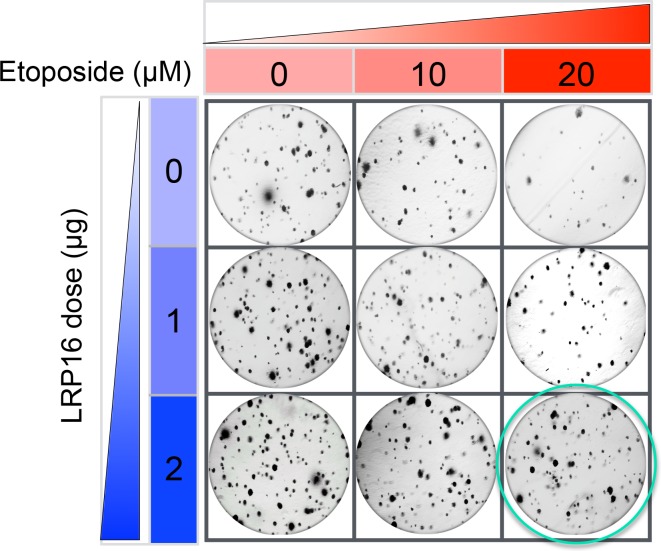


Secondly, the reader also had some questions about the Western Blots, namely whether there was any explanation why the sharp of the bands of p65 and IKK and their phosphorylated forms was dissimilar. We would like to clarify that in the published article, we ran identical batch of samples with the same quality, the same sequence and the same conditions to detect p65 and IKK and their phosphorylated forms and that these were run on different gels, thus explaining why there is some variance in the band shape.

Finally, the reader also noted that while the LRP16/MACROD1 signal is nicely present at 35 kDa (its predicted size), as illustrated well in this published paper (https://doi.org/10.3389/fmicb.2018.00020) it becomes clear that LRP16/MACROD1 runs at 28 kDa and that this is due to processing of the N-terminus. The reader focused on which antibody was used in our study or how this antibody was verified. The reader also suggested that it would be helpful to see at least one whole blot probed with this antibody, to see whether perhaps at 28 kDa a second band appears, corresponding to the processed form. Thus, our explanations for these questions are that, 1) LRP16/MACROD1 antibody (rabbit derived polyclonal antibody) used in this paper was prepared and produced by our laboratory. 2) about the verification of this antibody, in fact, we verified this antibody working well in our system, repeatedly. For instance, as shown below, in cells, we transfected different concentrations of LRP16/MACROD1 vector and empty vector, and then detected the LRP16/MACROD1 protein with this antibody, we found that with the concentration of the LRP16/MACROD1 vector increased, the LRP16/MACROD1 position showed stronger band. 3) about the LRP16/MACROD1 protein size we labelled in this paper, LRP16/MACROD1 was first cloned by our laboratory, according to its cDNA sequence, we also observed that LRP16/MACROD1 cDNA has two transcription start sites (TSS) within its open reading frame (ORF). Thus, we speculated that there may be different isoforms of LRP16/MACROD1 (long isoform, theoretical molecular weight 35kDa, short isoform, theoretical molecular weight 28kDa). In fact, we could detect two forms by our LRP16/MACROD1 antibody. As shown below, although LRP16/MACROD1 is predicted to exist in two different sizes of protein forms, there are not many studies on this gene, and its exact molecular weight is still not quite certain. Therefore, in our published paper, the molecular weight of LRP16/MACROD1 is based on the size of the commercialized proteins marker. Since the short isoform of LRP16/MACROD1 is the main form in multiple cell lines we used in this article, the display of LRP16/MACROD1 in our figures is the short isoform of LRP16/MACROD1. In order to be consistent with this published paper (https://doi.org/10.3389/fmicb.2018.00020), we declare that the LRP16/MACROD1 protein we exhibited in the published version is the short isoform of LRP16/MACROD1 signal as shown below (theoretical 28 kDa size).

**Figure 7:**
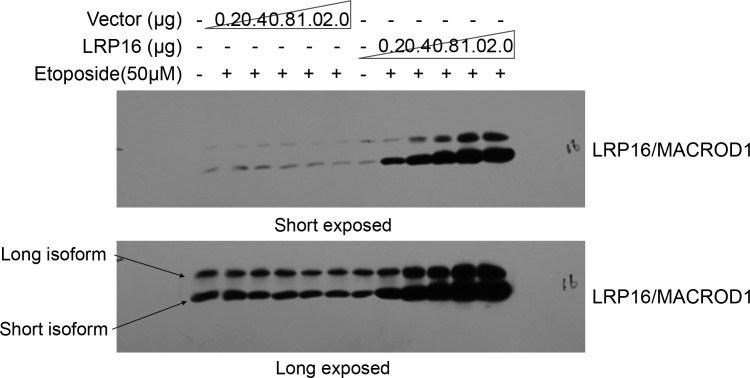


We apologize for our unintentional oversights and mistakes as mentioned above, inappropriate size label of LRP16/MACROD1 protein, and any inconvenience or confusion it may have caused.

The article has been corrected accordingly.

