# Supplementary material for: Blockade of the LRP16-PKR-NF-κB signaling axis sensitizes colorectal carcinoma cells to DNA-damaging cytotoxic therapy
Source: eLife. 2017 Aug 18;6:e27301. doi: 10.7554/eLife.27301 (PMC5562444; doi:10.7554/eLife.27301)
Supplement: Figure 1—source data 1. [file elife-27301-fig1-data1.docx]

**Figure 1—source data 1**

**LRP16 expression in tumor-adjacent tissues of the colorectal carcinoma (CRC) patient subgroups according to clinical pathological parameters**

| **Characteristic** | **All** | **Low LRP16**  **(*N* = 87)** | **High LRP16**  **(*N* = 115)** | **Comparison of LRP16 Levels, *P* Value** |
| --- | --- | --- | --- | --- |
| **Age (years)** | | | | |
| ≥ 50 | 136 | 63 | 73 | 0.1800 |
| < 50 | 66 | 24 | 42 |  |
| **Gender** | | | | |
| Male | 135 | 59 | 76 | 0.7960 |
| Female | 67 | 28 | 39 |  |
| **Histologically Differentiation** | | | | |
| Well differentiated | 41 | 22 | 19 | 0.1925 |
| Moderately differentiated | 99 | 37 | 62 |  |
| Poorly differentiated | 62 | 28 | 34 |  |
| **Tumor Grade** | | | | |
| I–II | 108 | 56 | 52 | **0.0069**** |
| III–IV | 94 | 31 | 63 |  |
| **T-stage** | | | | |
| T1, T2 | 37 | 22 | 15 | **0.0259*** |
| T3, T4 | 165 | 65 | 100 |  |
| **Lymph-Node Metastasis** | | | | |
| N0 | 120 | 59 | 61 | **0.0343*** |
| N1a, N1b, N1c | 82 | 28 | 54 |  |
| **Distant Metastasis** | | | | |
| M0 | 148 | 71 | 77 | **0.0198*** |
| M1a, M1b | 54 | 16 | 38 |  |

*P* value was determined with a Chi-squared (χ^2^) test.

* Parameters in bold, p < 0.05; **Parameters in bold, p < 0.01.
